# Safety, tolerability, pharmacokinetics and pharmacodynamics of GSK2239633, a CC-chemokine receptor 4 antagonist, in healthy male subjects: results from an open-label and from a randomised study

**DOI:** 10.1186/2050-6511-14-14

**Published:** 2013-02-28

**Authors:** Anthony Cahn, Simon Hodgson, Robert Wilson, Jonathan Robertson, Joanna Watson, Misba Beerahee, Steve C Hughes, Graeme Young, Rebecca Graves, David Hall, Sjoerd van Marle, Roberto Solari

**Affiliations:** 1 Medicines Discovery and Development, Gunnels Wood Road, Stevenage Herts SG1 2NY, UK; 2 GlaxoSmithKline, Stockley Park West, Uxbridge, Middlesex UB11 1BT, UK; 3 GlaxoSmithKline, Park Road, Ware SG12 0DP, UK; 4 PRA International, Stationsweg 163, Zuidlaren 9741 GP, the Netherlands

**Keywords:** GSK2239633, CCR4, Microdose, Healthy

## Abstract

**Background:**

The CC-chemokine receptor 4 (CCR4) is thought potentially to play a critical role in asthma pathogenesis due to its ability to recruit type 2 T-helper lymphocytes to the inflamed airways. Therefore, CCR4 provides an excellent target for anti-inflammatory therapy.

**Methods:**

The safety, tolerability, pharmacokinetics and pharmacodynamics of the CCR4 antagonist GSK2239633, N-(3-((3-(5-chlorothiophene-2-sulfonamido)-4-methoxy-1H-indazol-1-yl)methyl)benzyl)-2-hydroxy-2-methylpropanamide, were examined in healthy males. Two studies were performed: 1) an open-label, study in which six subjects received a single intravenous infusion of [^14^C]-GSK2239633 100 μg (10 kBq) (NCT01086462), and 2) a randomised, double-blind, placebo-controlled, cross-over, ascending dose study in which 24 subjects received single oral doses of GSK2239633 150–1500 mg (NCT01371812).

**Results:**

Following intravenous dosing, plasma GSK2239633 displayed rapid, bi-phasic distribution and slow terminal elimination (t_½_: 13.5 hours), suggesting that GSK2239633 was a low to moderate clearance drug. Following oral dosing, blood levels of GSK2239633 reached C_max_ rapidly (median t_max_: 1.0–1.5 hours). Estimated GSK2239633 bioavailability was low with a maximum value determined of only 16%. Food increased GSK2239633 systemic exposure (as assessed by AUC and C_max_). Increases in AUC and C_max_ were less than dose proportional. Adverse events were reported by three subjects (50%) following intravenous administration, and by 19 subjects (79%) following oral administration; most (46/47; 98%) events were mild/moderate in intensity. GSK2239633 1500 mg inhibited thymus- and activation-regulated chemokine-induced (TARC) actin polymerisation reaching a mean CCR4 occupancy of 74%.

**Conclusion:**

In conclusion, GSK2239633 was well-tolerated and capable of inhibiting TARC from activating the CCR4 receptor.

## Background

Allergic asthma is characterised by chronic inflammation of the airways, commonly triggered by environmental aeroallergens. This inflammatory process in asthma is characterised by inflammatory cell recruitment, increased mucus production, periodic airway smooth muscle contraction and vascular vasodilation [1-4]. Subsequently, Type 2 T helper (Th2) lymphocytes, and other cell types, are recruited from peripheral blood into the inflamed tissue where they produce cytokines that induce eosinophil recruitment, stimulate the production of allergen-specific immunoglobulin E by B-cells and increase the permeability of the endothelium to allow further recruitment of inflammatory cells [5-7].

One mode of Th2 lymphocyte recruitment to inflamed airways [8-11] is through the specific binding of the chemokines thymus- and activation-regulated chemokine (TARC) and monocyte-derived chemokine (MDC) to the CC-chemokine receptor 4 (CCR4) expressed on the surface of a subset of Th2 cells [12-17]. High levels of TARC and MDC have been detected in the lungs of patients with asthma following an allergen challenge [18,19], and high numbers of Th2 cells recovered in bronchial biopsies from patients with asthma have been found to be CCR4 positive [20-22]. As chemokine receptors play a key role in inflammatory processes, they provide excellent targets for anti-inflammatory therapy [23-26]. The targeting of CCR4 is believed to be a safe strategy as initial clinical studies with mogamulizumab, a humanised anti-CCR4 monoclonal antibody, have given no indication of an increase in the number of infections or any degree of immunosuppression related to mogamulizumab [27]. Further, there was no evidence of any CCR4-specific adverse clinical effects in patients with T-cell lymphomas treated with mogamulizumab [27].

GSK2239633 N-(3-((3-(5-chlorothiophene-2-sulfonamido)-4-methoxy-1H-indazol-1-yl)methyl)benzyl)-2-hydroxy-2-methylpropanamide, compound 7r in [28], is an allosteric antagonist of human CCR4 [29]. *In vitro*, GSK2239633 inhibited the binding of [^125^I]-TARC to human CCR4 with a pIC_50_ of 7.96 ± 0.11 and also inhibited TARC-induced increases in the F-actin content of isolated human CD4^+^ CCR4^+^ T-cells with a pA_2_ of 7.11 ± 0.29 [unpublished observations]. Conflicting pharmacokinetic profiles for GSK2239633 were obtained in the rat and dog, leading to variable predictions of the human pharmacokinetic profile. Therefore, before oral GSK2239633 was administered to humans for the first time, a Microdose Intravenous Study using a radio-labelled dose [30] of GSK2239633 was conducted in healthy subjects. This Microdose Intravenous Study revealed that plasma clearance of GSK2239633 was low to moderate or approximately 40% of liver blood flow when plasma clearance was converted to blood clearance. To obtain information at a potentially clinically relevant dose level, a study using single ascending oral doses of GSK2239633 was conducted in healthy subjects. Here we report the results obtained in both of these clinical studies.

## Methods

### Study population

All subjects provided signed and dated informed consent prior to screening. Local Ethics Review Committees provided approval for both studies (Microdose Intravenous Study: Independent Ethics Committee of the Foundation “Evaluation of the Ethics of Biomedical Research”, Assen, The Netherlands; Single Oral Dose Study: Medische Ethische ToetsingsCommissie, Stichting Beoordeling Ethik Biomedisch Onderzoek, Assen, The Netherlands) and they were conducted in accordance with Good Clinical Practice and the guiding principles of the 2008 Declaration of Helsinki [31].

#### Microdose intravenous study

Healthy subjects aged 18–50 years with a body mass index of 18.5–29.9 kg/m^2^ were eligible. Subjects had to be non-smokers or ex-smokers for a minimum of 6 months prior to screening and with a smoking history of <5 pack years. Exclusion criteria included positive testing for hepatitis B surface antigen, hepatitis C antibody and human immunodeficiency virus. Subjects unwilling to abstain from red wine, Seville oranges, grapefruit or grapefruit products 7 days prior to dosing were also ineligible.

#### Single oral dose study

Healthy subjects aged 18–65 years with a body mass index of 18.5–29.9 kg/m^2^ and a smoking history as described for the Microdose Intravenous Study were eligible. Key exclusion criteria were as for the Microdose Intravenous Study.

### Study design

#### Microdose intravenous study

This was an open-label, single-dose study conducted from 21 January 2010 to 18 February 2010 at PRA International, Zuidlaren, The Netherlands (GlaxoSmithKline protocol: CC4114041; Clinicaltrials.gov identifier: NCT01086462). Subjects received a single intravenous infusion of approximately 10 kBq [^14^C]-GSK2239633 100 μg over 15 minutes. Subjects attended a screening visit within 30 days prior to receiving the first dose of study medication. Subjects were admitted to the clinical unit on Day −1 and remained there until 48 hours post-dose. As this was the first time GSK2239633 100 μg had been administered to humans, dosing was staggered so that only one subject received the study medication on Day 1. As the study medication was well-tolerated, the remaining subjects were dosed the following day in a staggered dosing schedule (20-minute interval between dosing of the subjects). Subjects received a follow-up telephone call 4–10 days after the last dose of study medication.

#### Single oral dose study

This was a randomised, double-blind, placebo-controlled, cross-over, single ascending-dose study conducted from 29 March 2011 to 1 July 2011 at PRA International, Zuidlaren, the Netherlands (GlaxoSmithKline protocol: CC4114660; Clinicaltrials.gov identifier: NCT01371812). Subjects completed a screening visit within 28 days prior to receiving the first dose of study medication. Subjects were admitted to the clinical unit the day before each dosing session for baseline assessments that included a physical examination and clinical laboratory tests. Single ascending oral doses of GSK2239633 or placebo were administered to two interlocking and alternating cohorts (Additional file 1: Figure S1) (Cohort 1 and Cohort 2), each of which consisted of 12 male subjects randomised to receive either active or placebo (eight active: four placebo). The randomisation schedule was generated prior to the start of the study using validated internal software. Subjects from Cohort 1 underwent four dosing sessions; the starting dose of GSK2239633 was 150 mg, followed by 600 mg, 1200 mg and 1200 mg after eating the standard United States Food and Drug Administration (FDA) high fat/high caloric meal to assess any food effect. Subjects from Cohort 2 underwent three dosing sessions; the starting dose of GSK2239633 was 300 mg, followed by 900 mg and 1500 mg. As this was a first-time-in-human study, dosing was staggered over 2 days so that on Day 1 one subject received GSK2239633 and one subject received placebo at each dosing session in both cohorts (with the exception of the food effect dosing session for Cohort 1). On Day 2, the remaining subjects were dosed provided GSK2239633 was well-tolerated on Day 1. Subjects fasted for 10 hours before each dosing session, except those in last period of Cohort 1 who ate 30 minutes prior to administration of GSK2239633 1200 mg. For both cohorts, no food was permitted up to 4 hours after administration of study medication. During a 2-hour period (1 hour pre-dose until 1 hour post-dose), no water was allowed with the exception of that taken with the study medication (240–300 mL). After receiving randomised treatment, subjects underwent a period of observation and assessments for 3 days. They returned to the clinical unit, following a washout period of approximately 14 days, to receive their next randomised dose of study medication, with additional 3-day inpatient assessments. Subjects returned for a follow-up visit 10–14 days after their last dose of study medication.

### Dosing and sample collection

#### Microdose intravenous study

Subjects were dosed over 15 minutes with: 10 μg/mL GSK2239633 (^14^C-labelled) in a saline solution for infusion (0.9% w/v sodium chloride solution) containing 10% w/v (2-hydroxypropyl)-beta-cyclodextrin.

Blood samples for pharmacokinetic analysis of plasma total radioactivity and GSK2239633 were collected at screening, Day −1, pre-dose and at 5, 10, 15, 20, 30, 45 minutes and 1, 1.5, 1.75, 2.25, 3.25, 4.25, 6.25, 8.25, 12.25, 16.25, 18.25, 24.25, 30.25, 36.25 and 48.25 hours from start of the infusion. Urine samples were collected prior to dosing and then until 24 hours after the infusion ended.

#### Single oral dose study

Subjects were dosed with GSK2239633 as a capsule formulation (Swedish orange coloured opaque hard gelatin capsules) with a unit dosage strength of 150 mg. Subjects received between one and 10 capsules (depending on the dose level), which were swallowed with 240 mL of water (or up to 300 mL of water in total for the higher number of capsules). Subjects randomised to placebo in each dosing session received the same number of capsules as those randomised to active treatment for the same dosing session.

Blood samples were drawn for pharmacokinetic analysis pre-dose and at 5, 15 and 30 minutes and 1, 2, 3, 4, 8, 10, 24 and 48 hours post-dose. For pharmacodynamic analysis, blood was collected pre-dose and at 1, 4 and 24 hours post-dose. The pharmacodynamic analysis was only conducted for subjects in the fasted condition; no analysis was performed for the fed cohort. An aliquot of urine was collected pre-dose; after dosing, all urine was collected and pooled during a 24-hour interval.

### Pharmacokinetic analysis

#### Microdose intravenous study

The primary endpoints were maximum observed concentration (C_max_), area under the concentration-time curve from time 0 to last measurable concentration (AUC_0–t_), AUC from time 0 extrapolated to infinity (AUC_0–∞_) and terminal half-life (t_½_) of GSK2239633 and [^14^C]-radioactivity, apparent clearance (CL) and volume of distribution at steady state (V_ss_) of GSK2239633 and cumulative urinary excretion of total radioactivity for 24 hours post-dose. Total radioactivity was measured directly by accelerator mass spectrometry. Plasma GSK2239633 concentrations were determined using an internally validated analytical method by accelerator mass spectrometry (further details provided as Additional file 1 material).

Urine radioactivity levels were measured by liquid scintillation counting with an external standardisation method. The lower limit of quantification (LLQ) was 0.98 pg/mL for the plasma assay and 10 pg GSK2239633 equiv/mL for the total plasma radioactivity assay. The LLQ for GSK2239633 in urine was 5 μg GSK2239633 equiv. Pharmacokinetic parameters for each subject were derived from plasma GSK2239633 concentration-time profiles by non-compartmental analysis using WinNonlin Professional Edition Version 5.2 or above (Pharsight Corporation, Mountain View, USA). Maximum observed concentration, time to C_max_ (t_max_), AUC from time 0 to 48 hours post-dose (AUC_0–48_), AUC_0–t_, AUC_0–∞_, CL, t_½_, volume of distribution during terminal elimination phase (Vd) and V_ss_ were determined.

#### Single oral dose study

Blood concentrations of GSK2239633 were determined by an internally validated analytical method based on extraction from a dried blood spot disc by addition of methanol, followed by high performance liquid chromatography/tandem mass spectrometry. The LLQ of the assay was 10 ng/mL (further details provided as Additional file 1 material). Analysis and derivation of pharmacokinetic parameters were conducted as for the Microdose Intravenous Study.

### Safety and tolerability assessments

#### Microdose and single dose studies

The primary endpoints of the Single Oral Dose Study were adverse events and clinically relevant changes in safety parameters. Adverse events were recorded throughout both studies. For each event, the potential causal relationship with the study drug was assessed by the investigator. Other safety assessments in both studies included clinical laboratory tests (chemistry, haematology, urinalysis), vital signs, 12-lead electrocardiogram (ECG) and continuous cardiac telemetry.

### Pharmacodynamic analysis

#### Single oral dose study

Blood samples (9 volumes) were collected into a 3.8% sodium citrate solution (1 volume) and incubated for 15 minutes at room temperature with saturating concentrations of fluorescein isothiocyanate (FITC)-conjugated mouse anti-human CD4 antibody and non-inactivating phycoerythrin (PE)-conjugated mouse anti-human CCR4 antibody (BD Biosciences, Oxford, United Kingdom), or appropriate isotype control antibodies. The samples were then incubated for 30 minutes at 37°C. For pre-clinical studies, antagonists or vehicle were added at the beginning of this incubation. Following this, the blood cells were incubated for 15 seconds with varying concentrations of TARC (PeproTech EC, London, United Kingdom) before addition of 10 volumes of fluorescence-activated cell sorting (FACS) lysing solution (BD Biosciences, Oxford, United Kingdom). After 30 minutes, the blood cell suspension was centrifuged (500 *g* for 5 minutes) and resuspended in fresh FACS lysing solution for further 10 minutes to ensure complete red blood cell lysis. The cell suspensions were centrifuged (500 *g* for 5 minutes) again and washed twice by resuspending in phosphate buffered saline (PBS) solution and centrifuging at 500 *g* for 5 minutes. After incubating the cell suspensions for 15 minutes with lysophosphatidylcholine (100 μg/mL) and Alexa fluor 647 phalloidin (0.075 units/mL), the cells were recovered by centrifugation at 500 *g* for 5 minutes and resuspended in PBS. The F-actin content of the CD4^+^ CCR4^+^ lymphocytes in each sample was determined on a FACSCantoII flow cytometer by measuring the mean Alexa fluor 647 fluorescence intensity of 1,000 cells. This was expressed as a fraction of the Alexa fluor 647 fluorescence intensity of the CCR4^–^ lymphocytes in the same sample. The fractional occupancy of CCR4 (Ro) was then estimated by determining the dose-ratio (DR) from the change in effective concentration giving 50% of the maximal response (EC_50_) of the TARC concentration-response curve before and after dosing with GSK2239633 and using the formula Ro = (DR – 1)/DR [32].

### Statistical analysis

#### Microdose intravenous study

No formal sample size estimation was performed. As this was an exploratory study, no formal statistical hypotheses for safety, tolerability or pharmacokinetics were tested.

#### Single oral dose study

No statistical analysis was done to determine the sample size. There was no statistical analysis of safety parameters.

Dose proportionality was primarily evaluated based on C_max_, AUC_0–10_ and AUC_0–t_ using the power model. Each parameter was log_e_-transformed prior to analysis. Additionally, a mixed model was fitted to the dose-normalised pharmacokinetic parameters to compare each dose with the reference dose (GSK2239633 150 mg). The data were log_e_-transformed prior to analysis and the results were then back-transformed to calculate ratios between the doses. Food effect was assessed by performing a statistical analysis of C_max_, AUC_0–10_ and AUC_0–t_ after log_e_-transformation of the data. An analysis of variance (ANOVA) model was fitted along with 90% confidence intervals (CIs) by a mixed effects model, with fed/fasted condition as a fixed effect and subject as a random effect. Using data obtained in the Microdose Intravenous Study it was possible to make an estimation of GSK2239633 bioavailability following oral administration. For that, AUC_0–10_ was used as a comparison.

For the pharmacodynamic analysis, population estimates of the parameters, such as EC_50_, were derived using non-linear mixed effects models in NonMEM Version 7 (ICON Development Solutions, PA, USA) for all profiles generated. Analysis of the entire individual pharmacodynamic and pharmacokinetic datasets was conducted to derive mean EC_50_ estimates pre-dose and in the presence of GSK2239633 (each subject acted as their own control as their pre-dose data was compared with their post-dose data). Although not a direct method for formal calculation of Ro, this DR was used to give an estimate of Ro as described above.

## Results

### Subject disposition and demographics

#### Microdose intravenous study

Six male subjects were enrolled and completed the study. The population mean [range] age was 22.7 [20.0–26.0] years and mean [range] body mass index was 22.1 [19.3–24.6] kg/m^2^ (Table 1). All subjects were Caucasian.

**Table 1:** Summary of subjects demographic characteristics

**Demographics**	**Microdose intravenous study (n = 6)**	**Single oral dose study (n = 24)**
Age, years; Mean [range]	22.7 [20.0–26.0]	37.2 [20.0–65.0]
Sex; n (%)		
Male	6 (100%)	24 (100%)
Height, cm; Mean [range]	180.0 [172.0–187.0]	180.5 [163.0–197.0])
Weight, kg; Mean [range]	71.8 [60.1–85.5]	81.0 [65.7–106.0]
Body mass index, kg/m^2^; Mean [range]	22.1 [19.3–24.6]	24.9 [19.8–29.1]
Ethnicity; n (%)		
Hispanic or Latino	0	1 (4%)
Not Hispanic or Latino	6 (100%)	23 (96%)
Race; n (%)^a^		
White-White/Caucasian/European heritage	6 (100%)	20 (83%)
White-Arabic/North African heritage	0	1 (4%)
Asian-Central/South Asian heritage	0	1 (4%)
Asian-South East Asian heritage	0	2 (8%)

#### Single oral dose study

Twenty-four male subjects were enrolled and completed the study. The population mean [range] age was 37.2 [20.0–65.0] years and mean [range] body mass index was 24.9 [19.8–29.1] kg/m^2^ (Table 1). Twenty (83%) subjects were of Caucasian/European heritage, one (4%) subject was of Central/South Asian heritage, one (4%) subject was of South East Asian heritage and two (8%) subjects were of Arabic/North Africa heritage.

### Pharmacokinetics

#### Microdose intravenous study

Following infusion, the plasma pharmacokinetics of GSK2239633 and total plasma radioactivity showed a rapid bi-exponential distribution phase followed by a slow terminal elimination phase. Terminal elimination t_½_ values were 13.5 hours (95% CI: 9.6, 18.8) for GSK2239633 and 31.6 hours (95% CI: 25.4, 39.3) for total plasma radioactivity (Table 2). Values of AUC for GSK2239633 were half those obtained for total plasma radioactivity (AUC_0–48_: GSK2239633, 4.420 ng.hour/mL; total plasma radioactivity: 8.840 ng GSK2239633 equiv.hour/mL). The intrinsic plasma clearance (CL: 21.9 L/hour) was low to moderate. The observed Vss and Vd values of 119 L (95% CI: 78.4, 182.0) and 424 L (95% CI: 275.0, 654.0), respectively, for GSK2239633 were relatively high suggesting good distribution of GSK2239633 from the plasma compartment into tissues. However, a degree of caution should be taken when interpreting the apparent high distribution since there was evidence in several subjects of secondary peaks in the concentration-time profiles although data were sparse. The amount of radioactive drug-related material recovered in the urine accounted for approximately 20% of the administered dose.

**Table 2:** Summary of derived plasma GSK2239633 pharmacokinetic parameters in the microdose intravenous study

	**[**^**14**^**C]-GSK2239633 (n = 6)**	**Total plasma drug-related radioactivity (n = 6)**
Parameters	Geometric mean (95% CI)	Geometric mean (95% CI)*
C_max_ (ng/mL)	7.451 (6.114, 9.079)	8.380 (7.503, 9.359)
AUC_0–48_ (ng.hour/mL)	4.420 (3.515, 5.556)	8.840 (7.538, 10.368)
AUC_0–∞_ (ng.hour/mL)	4.577 (3.606, 5.810)	11.418 (9.004, 14.480)
t_½_ (hour)^1^	13.5 [8.0–21.2]	31.6 [25.1–43.0]
CL (L/hour)	21.9 (17.2, 27.7)	8.8 (6.9, 11.1)
V_ss_ (L)	119.0 (78.4, 182.0)	249.0 (200.0, 309.0)
Vd (L)	424.0 (275.0, 654.0)	399.0 (322.0, 494.0)

* Concentrations are in ng GSK2239633 equiv/mL and ng GSK2239633 equiv.hour/mL for C_max_ and AUC, respectively.

^1^ Median [range].

CI: confidence interval; C_max_: maximum observed concentration; AUC_0–48_: area under the concentration-time curve from time 0 to 48 hours post-dose; AUC_0–∞_: area under the concentration-time curve from time 0 extrapolated to infinity; t_½_: half-life; CL: apparent clearance; V_ss_: volume of distribution at steady state; Vd: volume of distribution during terminal elimination phase.

#### Single oral dose study

Absorption of GSK2239633 was rapid with C_max_ at 1.0–1.5 hours across the dose range (Table 3). Values of t_½_ could only be calculated for one subject after the 150 mg and the 1200 mg (fed) doses, and two subjects after the 1200 mg (fed) dose as blood concentrations of GSK2239633 were too erratic and low during the terminal phase for other subjects; for these three subjects, t_½_ values ranged from 2.9 to 28.3 hours. Generally, both AUC (Additional file 1: Figure S2A) and C_max_ (Additional file 1: Figure S2B) increased with GSK2239633 dose. Results from the power model analysis indicated a less than dose proportional increase for AUC_0–10_ (adjusted mean slope: 0.61; 90% CI: 0.54, 0.68) and C_max_ (adjusted mean slope: 0.76; 90% CI: 0.63, 0.89) as the 90% CIs did not contain unity (Additional file 1: Table S1). Dose proportionality results were supported by mixed model analysis as assessed by comparison with the GSK2239633 150 mg dose level. Mean C_max_ and AUC_0–t_ for GSK2239633 following a dose of 1200 mg were 695 ng/mL and 2330 ng.hour/mL, respectively; these increased to 1410 ng/mL and 6520 ng.hour/mL when the study medication was administered after a standard FDA high fat/high caloric meal. The analysis of the food effect for GSK2239633 showed an increase of 208% in AUC_0–10_, 180% in AUC_0–t_ and 103% in C_max_ (GSK2239633 1200 mg fed:fasted) in the fed state; all these differences achieved statistical significance (Supplemental Table S2). In addition to the increases observed for C_max_ and AUC parameters, absorption of GSK2239633 was more protracted in the fed condition with a median t_max_ of 3.0 hours. GSK2239633 bioavailability in the fasted state ranged from 12–14% for the two lowest doses studied (GSK2239633 150 mg and 300 mg). For GSK2239633 600 mg and above, estimated bioavailability decreased ranging from 5–9%. In the fed state, GSK2239633 estimated bioavailability increased to approximately 16%.

**Table 3:** Summary of derived blood GSK2239633 pharmacokinetic parameters in the single oral dose study (geometric mean, 95% confidence interval)

	**GSK2239633 Dose**						
**Parameter**	**150 mg(n = 8)**	**300 mg (n = 8)**	**600 mg (n = 8)**	**900 mg (n = 8)**	**1200 mg (n = 8)**	**1500 mg (n = 8)**	**1200 mg (Fed) (n = 8)**
AUC_0–10_ (ng.hour/mL)	534 (430, 663)	923 (695, 1227)	1210 (1055, 1393)	2060 (1552, 2741)	1520 (1175, 1963)	2560 (2026, 3234)	4670 (3783, 5769)
AUC_0–t_ (ng.hour/mL)	718 (518, 995)	1560 (971, 2511)	1970 (1563, 2492)	3110 (2242, 4324)	2330 (1499, 3611)	4150 (3121, 5528)	6520 (4264, 9980)
AUC_0–∞_ (ng.hour/mL)	420	ND	ND	ND	ND	ND	5180 (3101, 8654)
%AUCex (%)	13.7	ND	ND	ND	ND	ND	9.4 (3.6, 24.8)
C_max_ (ng/mL)	178 (124, 256)	353 (237, 526)	538 (391, 739)	869 (622, 1215)	695 (476, 1016)	1210 (778, 1889)	1410 (1127, 1761)
t_max_ (hour)^1^	1.5 [1.0–3.0]	1.0 [1.0–3.0]	1.0 [0.5–2.0]	1.5 [0.5–3.0]	1.0 [0.5–3.0]	1.0 [0.5–4.0]	3.0 [0.6–4.0]

^1^ Median [range].

AUC_0–10_: area under the concentration-time curve from time 0 to 10 hours post-dose; AUC_0–t_: AUC from time 0 to last measurable concentration; AUC_0–∞_: AUC from time 0 extrapolated to infinity; ND: not determined; %AUCex: percentage of AUC_0–∞_ obtained by extrapolation; C_max_: maximum observed concentration; t_max_: time to C_max_.

### Safety and tolerability

#### Microdose intravenous study

Adverse events were reported by three of the six (50%) study subjects. The events reported were abdominal discomfort (n = 1), diarrhoea (n = 1), rhinitis (n = 1) and headache (n = 1); all were mild, transient and had resolved at follow-up. The episode of diarrhoea was the only event judged to be possibly drug-related by the investigator. There were no clinically significant abnormalities in clinical laboratory results, physical exam, vital signs, 12-lead ECG parameters or continuous cardiac telemetry.

#### Single oral dose study

Nineteen of the 24 (79%) subjects reported adverse events (Table 4). There was no dose response in the incidence of adverse events across dose and treatment groups. Forty-six of the 47 (98%) events were graded as mild or moderate; none was graded as severe. One subject had a skin mole at screening that was subsequently excised and found to be a malignant melanoma. This was reported as an adverse event. The most frequently reported adverse events were headache and diarrhoea. Six subjects experienced events judged to be drug-related by the investigator, the most frequent of which was diarrhoea reported by three (13%) subjects (GSK2239633 600 mg: one; GSK2239633 1500 mg: two; placebo: one). Other events judged to be drug-related were abdominal pain, chest discomfort, headache, oral herpes and somnolence (one subject each). All adverse events resolved by the end of the study except for one episode of joint injury and one episode of folliculitis. No trends were detected in changes from baseline for clinical laboratory test values. The investigator judged there to be no clinically significant abnormalities in vital sign, 12-lead ECG parameters or continuous cardiac telemetry.

**Table 4:** Summary of the adverse events reported by two or more subjects in the single oral dose study

		**GSK2239633**								
	**Placebo (n = 24) n (%)**	**150 mg (n = 8) n (%)**	**300 mg (n = 8) n (%)**	**600 mg (n = 8) n (%)**	**900 mg (n = 8) n (%)**	**1200 mg (n = 8) n (%)**	**1500 mg (n = 8) n (%)**	**1200 mg (Fed) (n = 8) n (%)**	**Placebo (Fed) (n = 4) n (%)**	**Total (n = 24) n (%)**
Any event	9 (38)	4 (50)	5 (63)	5 (63)	1 (13)	2 (25)	3 (38)	2 (25)	1 (25)	19 (79)
Headache	3 (13)	2 (25)	0	0	1 (13)	1 (13)	1 (13)	0	0	5 (21)
Diarrhoea	2 (8)	0	2 (25)	1 (13)	1 (13)	0	2 (25)	0	0	4 (17)
Rhinitis	1 (4)	1 (13)	0	0	0	0	1 (13)	0	0	2 (8)
Abdominal pain	0	0	0	0	0	0	1 (13)	0	0	1 (4)

### Pharmacodynamics

#### Single oral dose study

**Figure 1:**
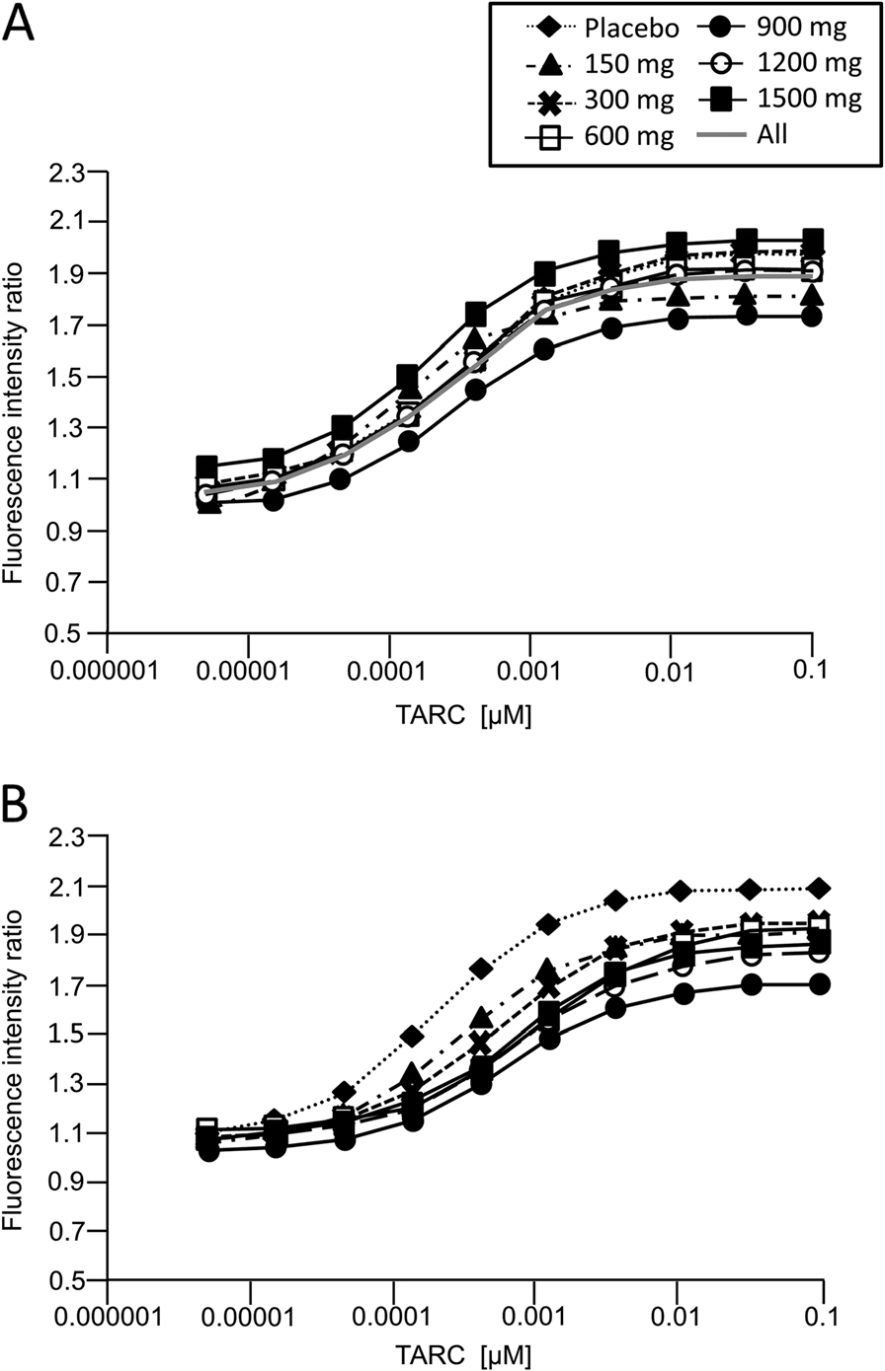
**Population fits per dose level for pre-dose (Panel A) and 1 hour post-dose (Panel B) in the Single Oral Dose Study.** Following single oral dosing of GSK2239633, blood samples were collected from subjects and stimulated with TARC as described. Formation of F-actin was determined following staining with Alexa fluor 647 phalloidin and analysis with a FACSCantoII flow cytometer by measuring the mean Alexa fluor 647 fluorescence intensity of 1,000 cells. The ratio of F-actin formation in CD4^+^ CCR4^+^ and CD4^+^ CCR4^-^ T-cells was calculated, and the fractional receptor occupancy of CCR4 (Ro) was then determined by estimating a dose-ratio (DR) from the change in effective concentration giving 50% of the maximal response (EC_50_) of the TARC concentration-response curve before and after dosing with GSK2239633 and using the formula Ro = (DR – 1)/DR.

**Figure 2:**
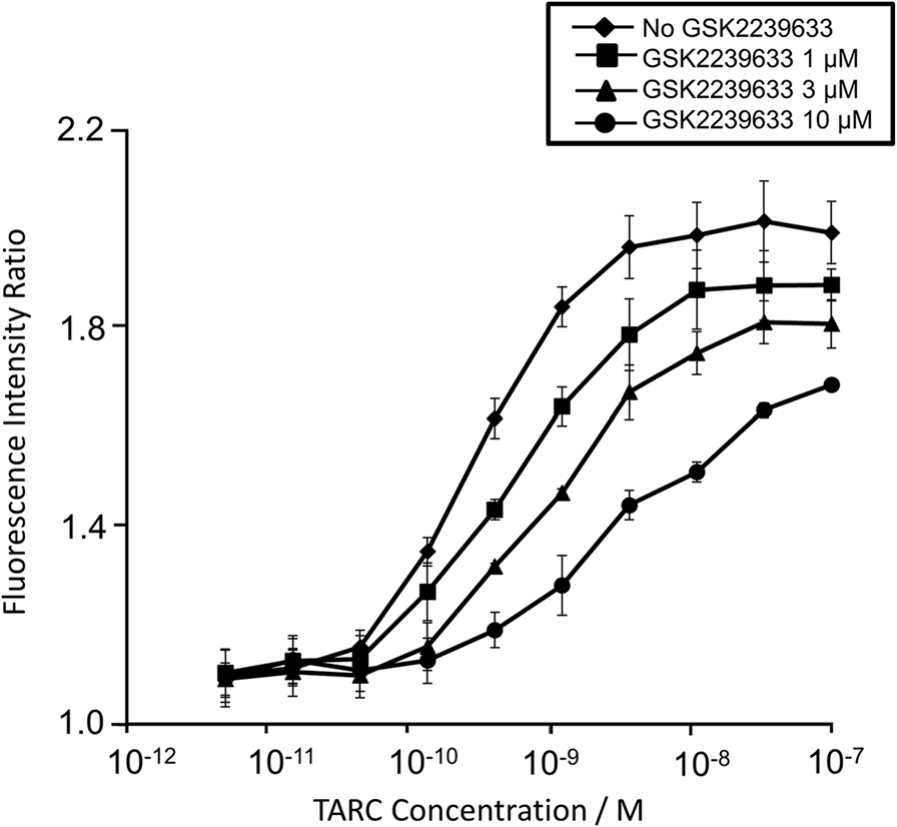
**Thymus- and activation-regulated chemokine-induced increases in F-actin content of CD4**^**+ **^**CCR4**^**+ **^**T-cells in whole human blood in the absence or presence of GSK2239633 at 1 μM, 3 μM or 10 μM.** The data presented are the mean of independent determinations in three donors. Error bars represent standard error of the mean.

Dose–response curves for the relative increase in filamentous actin (F-actin) content of CD4^+^ CCR4^+^ cells in response to TARC showed that, although highly variable, CCR4 inhibition was evident from GSK2239633 150 mg to 1500 mg (Figure 1). For comparison, the results from *in vitro* studies assessing the effect of GSK2239633 (1–10 μM) on TARC-induced increases in F-actin content of CD4^+^ CCR4^+^ T-cells are also presented (Figure 2). At the GSK2239633 1500 mg dose, the calculated mean level of CCR4 inhibition equated to a predicted Ro of approximately 74% at 1 hour after dosing. Predicted Ro levels decreased over time following the blood pharmacokinetic profile, which showed an initial rapid peak in blood GSK2239633 exposure followed by a rapid decline to much lower levels 4–8 hours post-dose. The Ro estimates for each dose group at 1 and 4 hours post-dose are presented in Table 5. Receptor occupancy estimates at 4 hours post-dose were more variable than those at 1 hour post-dose and therefore, difficult to interpret. Although, this was not unexpected given the low blood exposure of GSK2239633 at the 4 hours post-dose time-point. Blood from placebo subjects, analysed in the same way and at the same time-points, did not show any shifts in the response curves, which would indicate CCR4 inhibition, when the post-dose curves were compared with the pre-dose curves. The placebo data also revealed the inherent variability in the technique as shown by the approximately 3-fold range in variability for the derived EC_50_ of TARC (pre-dose = 0.34 nM; all data = 0.1–0.34 nM).

**Table 5:** Changes in estimated receptor occupancy in the presence of GSK2239633 in the single oral dose study

**GSK22939633 dose**	**Parameter**^**1,2**^	**Post-dose time point**	
		**1 hour**	4 hours**
150 mg	Ro (95% CI) (%)	63 (NC)	53 (NC)
300 mg	Ro (95% CI) (%)	37 (29, 54)	9 (0, 45)
600 mg	Ro (95% CI) (%)	72 (71, 75)	14 (13, 14)
900 mg	Ro (95% CI) (%)	55 (50, 58)	42 (0, 56)
1200 mg	Ro (95% CI) (%)	64 (60, 67)	0 (0, 1)
1500 mg	Ro (95% CI) (%)	74 (62, 79)	61 (52, 67)

^1^ Ro: CCR4 occupancy by GSK2239633 derived from the EC_50_ ratio (DR) for each dose group divided by the pre-dose EC_50_ and converted to an estimate of CCR4 occupancy (DR-1/DR).

^2^ 95% CI: calculated from the 95% confidence interval (when available) of the modelled EC_50_.

** Data generated at 4 hours post-dose with GSK2239633 was highly variable.

Ro: receptor occupancy; CI: confidence interval; NC: not calculated, insufficient data to generate a measure of variability.

## Discussion

In the Microdose Intravenous Study, the clearance of GSK2239633 was low to moderate (approximately 40% of liver blood flow) with a proportion of the drug appearing to be well distributed based on the long terminal elimination rate observed, albeit at relatively low levels and with signs of secondary input. Prior to the conduct of the Single Oral Dose Study, the systemic bioavailability of GSK2239633 in humans was predicted to be, at best, approximately 70%, based on results of the Microdose Intravenous Study and those obtained in the pre-clinical studies. This assumed that bioavailability was limited only by first pass extraction equivalent to the systemic clearance measured in the Microdose Intravenous Study. However, findings in the Single Oral Dose Study showed that this was an overestimate (see below). Unchanged GSK2239633 accounted for only 50% of the total plasma radioactivity following intravenous administration with the remaining circulating drug-related material comprised of one or more metabolites. A glucuronide conjugate was identified as a major component of the metabolised fraction through a pooled plasma analysis.

The blood exposure of GSK2239633 achieved in the Single Oral Dose Study was substantially lower than that expected based on the high oral bioavailability obtained in several pre-clinical species; however, it may be consistent with the physicochemical properties of the molecule, high molecular weight (549) and low solubility (0.02 mg/mL), and could explain the limited absorption window observed in humans. Gastric motility and the presence of food in the stomach could influence the systemic exposure profile of GSK2239633 in the manner observed in this first in man oral study. The food effect analysis supports this hypothesis and administration of GSK2239633 1200 mg in the fed state led to statistically significant increases in C_max_ (103%) and AUC parameters (AUC_0–10_: 208%; AUC_0–t_: 180%) compared with the fasted state. However, other factors, such as solubility, cannot be excluded. In addition, in the fed state, t_max_ was delayed by approximately 1.5 hours compared with the fasted state. The estimated bioavailability of GSK2239633 was considerably lower than predicted, achieving a maximum of only approximately 16% (either fasted or fed). Therefore, GSK2239633 exposure in the blood was notably lower than that required to explore a full pharmacodynamic response, which was reflected in the low estimated Ro observed for the actin polymerisation pharmacodynamic endpoint.

In these early studies, GSK2239633 had a satisfactory safety and tolerability profile in healthy subjects. No dose-limiting toxicity or maximum tolerated dose was identified. There was no relationship in the frequency or severity of adverse events with increasing doses of GSK2239633. The anti-CCR4 antibody, mogamulizumab, has been tested in clinical studies, although the study population for this was patients with relapsed CCR4^+^ adult T-cell lymphomas and other peripheral T-cell lymphomas [27]. Mogamulizumab has thus far been shown to be well-tolerated in that study population and none of the adverse events reported were specific to inhibition of CCR4 [27].

Pharmacodynamic analysis in the Single Oral Dose Study revealed that GSK2239633 inhibited TARC-induced increases in the F-actin content of CCR4^+^ T cells in human whole blood. Although, even at the highest dose (1500 mg), the magnitude of inhibition was low (the potency of TARC decreased only 4-fold indicating that the mean estimated receptor occupancy was 74% at 1 hour post-dose) and relatively short-acting (reflecting the rapid reduction of drug concentration and inhibition in the blood at 4 hours post-dose). GSK2239633 failed to achieve the minimum target level of CCR4 inhibition in the blood (≥90% at peak and 50% at trough), and there was no indication of an extended duration of action (prolonged pharmacodynamic response in the absence of pharmacokinetic exposure) in whole blood *ex vivo*. In this study, we were unable to obtain systemic exposure of GSK2239633 high enough to inhibit CCR4 by more than 80% as measured in the whole blood CCR4 pharmacodynamic assay. This would substantially limit the degree to which we would be able to assess the blockade of CCR4 for clinical benefit in further studies.

CC-Chemokine receptor 4 is potentially involved in the pathogenesis of allergic diseases due to its involvement in the pathways leading to the recruitment of Th2 cells to the sites of allergen exposure [22,33]. A number of small-molecule CCR4 antagonists have shown promising results in various animal models of inflammation, such as reduction in ovalbumin-induced ear swelling in mice [34], inhibition of ovalbumin-induced airway inflammation in guinea pigs [35] and a reduction in the recruitment of Th2 cells to the lungs in a mouse model of ovalbumin-induced airway allergy [36]. However, no small molecules have progressed to clinical studies thus far [2,26], which may be due, in part, to poor oral exposure noted in pre-clinical animal models with some of the small molecules reported. A further general challenge with chemokines often speculated upon is the potential for redundancy within the chemokine system and alteration of function during evolution [2,37]. Results from some studies [38-40] indicate that chemokine receptors are also a good target for adjuvant discovery, in particular CCR4, as this receptor is expressed by regulatory T cells, a subset of T cells which normally functions in the down-regulation of immune responses induced by dendritic cells [41]. One of these studies identified CCR4 antagonists acting as adjuvants for both cellular and humoral immune responses.

## Conclusions

Results obtained from these early studies conducted in healthy subjects, indicate that GSK2239633 was generally safe and well tolerated. GSK2239633 exhibited low and saturable systemic exposures and at the highest dose level of 1500 mg the peak inhibition of CCR4 by GSK2239633 in the blood (at 1 hour) was below 80% and less than 50% by 4 hours post-dose. Based on the low exposure and target engagement in blood, this molecule is not considered suitable for further development for an asthma indication at this time.

## Abbreviations

ANOVA: Analysis of variance;AUC0–48: Area under the concentration-time curve from time 0 to 48 hours post-dose;AUC0–t: AUC from time 0 to last measurable concentration;AUC0–∞: AUC from time 0 extrapolated to infinity;CCR4: CC-chemokine receptor 4;CI: Confidence Intervals;CL: Apparent clearance;Cmax: Maximum observed concentration;DR: Dose-Ratio;EC50: Effective concentration giving 50% of the maximal response;FACS: Fluorescence-activated cell sorting;FDA: United states food and drug administration;FITC: Fluorescein isothiocyanate;LLQ: Lower limit of quantification;MDC: Monocyte-derived chemokine;PBS: Phosphate buffered saline;PE: Phycoerythrin;Ro: Fractional occupancy;TARC: Thymus- and activation-regulated chemokine;t½: Terminal half-life;tmax: Time to C_max_;Th2: Type 2 T helper;Vd: Volume of distribution during terminal elimination phase;Vss: Volume of distribution at steady state

## Competing interests

Anthony Cahn, Simon Hodgson, Robert Wilson, Jonathan Robertson, Joanna Watson, Misba Beerahee, Steve C. Hughes, Graeme Young, Rebecca Graves, David Hall and Roberto Solari are GlaxoSmithKline employees. Sjoerd van Marle has no competing interests.

## Authors’ contributions

AC, RW, JR and MB participated in the conception and design of the study, and in the analysis and interpretation of the data. JW and SCH participated in the conception and design of the study, and in the acquisition, analysis and interpretation of the data. SH, DH and SVM took part in the acquisition, analysis and interpretation of the data. GY, RG and RS contributed in the analysis and interpretation of the data. All authors have made critical revisions of draft versions of the manuscript and approved the final manuscript.

## Pre-publication history

The pre-publication history for this paper can be accessed here:

http://www.biomedcentral.com/2050-6511/14/14/prepub

## Supplementary Material

Additional file 1 **Safety, tolerability, pharmacokinetics and pharmacodynamics of GSK2239633, a CC-chemokine receptor 4 antagonist, in healthy male subjects.** Detailed information of the pharmacokinetic assays, 2 Tables, 2 Figures and figure legends. Click here for file

## References

[B1] Finiasz M Otero C Bezrodnik L Fink S The role of cytokines in atopic asthma Curr Med Chem 2011 18 1476 1487 10.2174/092986711795328346 21428894

[B2] Pease JE Targeting Chemokine receptors in allergic disease Biochem J 2011 434 11 24 10.1042/BJ20101132 21269275

[B3] Azzawi M Bradley B Jeffery PK Frew AJ Wardlaw AJ Knowles G Assoufi B Collins JV Durham S Kay AB Identification of activated T lymphocytes and eosinophils in bronchial biopsies in stable atopic asthma Am Rev Respir Dis 1990 142 1407 1413 2252260 10.1164/ajrccm/142.6_Pt_1.1407

[B4] Fanta CH Asthma N Engl J Med 2009 360 1002 1014 10.1056/NEJMra0804579 19264689

[B5] Hamid Q Tulic M Immunobiology of asthma Annu Rev Physiol 2009 71 489 507 10.1146/annurev.physiol.010908.163200 19575684

[B6] Long AA Immunomodulators in the treatment of asthma Allergy Asthma Proc 2009 30 109 119 10.2500/aap.2009.30.3203 19463201

[B7] Minai-Fleminger Y Levi-Schaffer F Mast cells and eosinophils: the two key effector cells in allergic inflammation Inflamm Res 2009 58 631 638 10.1007/s00011-009-0042-6 19440657

[B8] Vestergaard C Yoneyama H Murai M Nakamura K Tamaki K Terashima Y Imai T Yoshie O Irimura T Mizutani H Matsushima K Overproduction of Th2-specific Chemokines in NC/Nga mice exhibiting atopic dermatitis-like lesions J Clin Invest 1999 104 1097 1157 10.1172/JCI7613 10525048 PMC408579

[B9] Vestergaard C Bang K Gesser B Yoneyama H Matsushima K Larsen CG A Th2 Chemokine, TARC, produced by keratinocytes may recruit CLA + CCR4+ lymphocytes into lesional atopic dermatitis skin J Invest Dermatol 2000 115 640 646 10.1046/j.1523-1747.2000.00115.x 10998136

[B10] Sekiya T Miyamasu M Imanishi M Yamada H Nakajima T Yamaguchi M Fujisawa T Pawankar R Sano Y Ohta K Ishii A Morita Y Yamamoto K Matsushima K Yoshie O Hirai K Inducible expression of a Th2-type CC Chemokine thymus- and activation-regulated Chemokine by human bronchial epithelial cells J Immunol 2000 165 2205 2213 10925308 10.4049/jimmunol.165.4.2205

[B11] Zheng X Nakamura K Furukawa H Nishibu A Takahashi M Tojo M Kaneko F Kakinuma T Tamaki K Demonstration of TARC and CCR4 mRNA expression and distribution using in situ RT-PCR in the lesional skin of atopic dermatitis J Dermatol 2003 30 26 32 12598706 10.1111/j.1346-8138.2003.tb00329.x

[B12] Liu YJ Thymic stromal lymphopoietin: master switch for allergic inflammation J Exp Med 2006 203 269 273 10.1084/jem.20051745 16432252 PMC2118215

[B13] Imai T Baba M Nishimura M Kakizaki M Takagi S Yoshie O The T cell-directed CC Chemokine TARC is a highly specific biological ligand for CC Chemokine receptor 4 J Biol Chem 1997 272 15036 15042 10.1074/jbc.272.23.15036 9169480

[B14] Imai T Chantry D Raport CJ Wood CL Nishimura M Godiska R Yoshie O Gray PW Macrophage-derived Chemokine is a functional ligand for the CC Chemokine receptor 4 J Biol Chem 1998 273 1764 1768 10.1074/jbc.273.3.1764 9430724

[B15] Imai T Nagira M Takagi S Kakizaki M Nishimura M Wang J Gray PW Matsushima K Yoshie O Selective recruitment of CCR4-bearing Th2 cells toward antigen-presenting cells by the CC Chemokines thymus and activation-regulated Chemokine and macrophage-derived Chemokine Int Immunol 1999 11 81 88 10.1093/intimm/11.1.81 10050676

[B16] Sallusto F Lenig D Mackay CR Lanzavecchia A Flexible programs of Chemokine receptor expression on human polarized T helper 1 and 2 lymphocytes J Exp Med 1998 187 875 883 10.1084/jem.187.6.875 9500790 PMC2212187

[B17] D’Ambrosio D Iellem A Bonecchi R Mazzeo D Sozzani S Mantovani A Sinigaglia F Selective up-regulation of Chemokine receptors CCR4 and CCR8 upon activation of polarized human type 2 Th cells J Immunol 1998 161 5111 5115 9820476

[B18] Bochner BS Hudson SA Xiao HQ Liu MC Release of both CCR4-active and CXCR3-active Chemokines during human allergic pulmonary late-phase reactions J Allergy Clin Immunol 2003 112 930 934 10.1016/j.jaci.2003.08.012 14610482

[B19] Pilette C Francis JN Till SJ Durham SR CCR4 Ligands are up-regulated in the airways of atopic asthmatics after segmental allergen challenge Eur Respir J 2004 23 876 884 10.1183/09031936.04.00102504 15219001

[B20] Panina-Bordignon P Papi A Mariani M Di Lucia P Casoni G Bellettato C Buonsanti C Miotto D Mapp C Villa A Arrigoni G Fabbri LM Sinigaglia F The C-C Chemokine receptors CCR4 and CCR8 identify airway T cells of allergen-challenged atopic asthmatics J Clin Invest 2001 107 1357 1364 10.1172/JCI12655 11390417 PMC209325

[B21] Morgan AJ Symon FA Berry MA Pavord ID Corrigan CJ Wardlaw AJ IL-4-expressing bronchoalveolar T cells from asthmatic and healthy subjects preferentially express CCR 3 and CCR 4 J Allergy Clin Immunol 2005 116 594 600 10.1016/j.jaci.2005.03.052 16159629

[B22] Vijayanand P Durkin K Hartmann G Morjaria J Seumois G Staples KJ Hall D Bessant C Bartholomew M Howarth PH Friedmann PS Djukanovic R Chemokine receptor 4 plays a key role in T cell recruitment into the airways of asthmatic patients J Immunol 2010 184 4568 4574 10.4049/jimmunol.0901342 20237293

[B23] Barnes PJ Immunology of asthma and chronic obstructive pulmonary disease Nat Rev Immunol 2008 8 183 192 10.1038/nri2254 18274560

[B24] Barnes PJ The cytokine network in asthma and chronic obstructive pulmonary disease J Clin Invest 2008 118 3546 3556 10.1172/JCI36130 18982161 PMC2575722

[B25] Donnelly LE Barnes PJ Chemokine receptors as therapeutic targets in chronic obstructive pulmonary disease Trends Pharmacol Sci 2006 27 546 553 10.1016/j.tips.2006.08.001 16911834

[B26] Hall D Ford A Hodgson S Therapeutic potential of CCR4 antagonists New drugs and targets for asthma and COPD. Volume 39 2010 Karger: Prog Respir Res Basel 161 165

[B27] Yamamoto K Utsunomiya A Tobinai K Tsukasaki K Uike N Uozumi K Yamaguchi K Yamada Y Hanada S Tamura K Nakamura S Inagaki H Ohshima K Kiyoi H Ishida T Matsushima K Akinaga S Ogura M Tomonaga M Ueda R Phase I study of KW-0761, a defucosylated humanized anti-CCR4 antibody, in relapsed patients with adult T-cell leukemia-lymphoma and peripheral T-cell lymphoma J Clin Oncol 2010 28 1591 1598 10.1200/JCO.2009.25.3575 20177026

[B28] Procopiou PA Ford AJ Graves RH Hall DA Hodgson ST Lacroix YM Needham D Slack RJ Lead optimisation of the N1 substituent of a novel series of indazole arylsulfonamides as CCR4 antagonists and identification of a candidate for clinical investigation Bioorg Med Chem Lett 2012 22 2730 2733 10.1016/j.bmcl.2012.02.104 22437117

[B29] Hodgson ST Lacroix YML Procopiou PA US patent 2010216860A1 2010: http://www.google.co.uk/patents?hl=en&lr=&vid=USPATAPP12711283&id=5OrUAAAAEBAJ&oi=fnd&dq=hodgson+patent+ccr4&printsec=abstract#v=onepage&q=hodgson%20patent%20ccr4&f=false

[B30] ICH M3 (R2) - guideline on nonclinical safety studies for the conduct of human clinical trials and marketing authorization for pharmaceuticals http://www.ema.europa.eu/docs/en_GB/document_library/Scientific_guideline/2009/09/WC500002941.pdf 20349552

[B31] World Medical Association Declaration of Helsinki - ethical principles for medical research involving human subjectshttp://www.wma.net/en/30publications/10policies/b3

[B32] Paton WDM A theory of drug action based on the rate of drug-receptor combination Proc R Soc Lond B 1961 154 21 69 10.1098/rspb.1961.0020

[B33] Banfield G Watanabe H Scadding G Jacobson MR Till SJ Hall DA Robinson DS Lloyd CM Nouri-Aria KT Durham SR CC Chemokine receptor 4 (CCR4) in human allergen-induced late nasal responses Allergy 2010 65 1126 1133 20148806 10.1111/j.1398-9995.2010.02327.x PMC3380530

[B34] Nakagami Y Kawashima K Yonekubo K Etori M Jojima T Miyazaki S Sawamura R Hirahara K Nara F Yamashita M Novel CC Chemokine receptor 4 antagonist RS-1154 inhibits ovalbumin-induced ear swelling in mice Eur J Pharmacol 2009 624 38 44 10.1016/j.ejphar.2009.09.058 19818758

[B35] Nakagami Y Kawase Y Yonekubo K Nosaka E Etori M Takahashi S Takagi N Fukuda T Kuribayashi T Nara F Yamashita M RS-1748, a novel CC Chemokine receptor 4 antagonist, inhibits ovalbumin-induced airway inflammation in guinea pigs Biol Pharm Bull 2010 33 1067 1079 10.1248/bpb.33.1067 20522980

[B36] Sato T Komai M Iwase M Kobayashi K Tahara H Ohshima E Arai H Miki I Inhibitory effect of the new orally active CCR4 antagonist K327 on CCR4 + CD4+ T cell migration into the lung of mice with ovalbumin-induced lung allergic inflammation Pharmacology 2009 84 171 182 10.1159/000235748 19713720

[B37] Catley MC Coote J Bari M Tomlinson KL Monoclonal antibodies for the treatment of asthma Pharmacol Ther 2011 132 333 351 10.1016/j.pharmthera.2011.09.005 21944943

[B38] Bayry J Tchilian EZ Davies MN Forbes EK Draper SJ Kaveri SV Hill AV Kazatchkine MD Beverley PC Flower DR Tough DF In silico identified CCR4 antagonists target regulatory T cells and exert adjuvant activity in vaccination Proc Natl Acad Sci USA 2008 105 10221 10226 10.1073/pnas.0803453105 18621704 PMC2481334

[B39] Davies MN Bayry J Tchilian EZ Vani J Shaila MS Forbes EK Draper SJ Beverley PC Tough DF Flower DR Toward the discovery of vaccine adjuvants: coupling in silico screening and *in vitro* analysis of antagonist binding to human and mouse CCR4 receptors PLoS One 2009 4 e8084 10.1371/journal.pone.0008084 20011659 PMC2787246

[B40] Pere H Montier Y Bayry J Quintin-Colonna F Merillon N Dransart E Badoual C Gey A Ravel P Marcheteau E Batteux F Sandoval F Adotevi O Chiu C Garcia S Tanchot C Lone YC Ferreira LC Nelson BH Hanahan D Fridman WH Johannes L Tartour E A CCR4 antagonist combined with vaccines induces antigen-specific CD8+ T cells and tumor immunity against self antigens Blood 2011 118 4853 4862 10.1182/blood-2011-01-329656 21908423

[B41] Miyara M Sakaguchi S Natural regulatory T cells: mechanisms of suppression Trends Mol Med 2007 13 108 116 10.1016/j.molmed.2007.01.003 17257897

