# Supplementary material for: Safety, tolerability, pharmacokinetics and pharmacodynamics of GSK2239633, a CC-chemokine receptor 4 antagonist, in healthy male subjects: results from an open-label and from a randomised study
Source: BMC Pharmacol Toxicol. 2013 Feb 28;14:14. doi: 10.1186/2050-6511-14-14 (PMC3599276; doi:10.1186/2050-6511-14-14)

Safety, tolerability, pharmacokinetics and pharmacodynamics of GSK2239633, a CC-chemokine receptor 4 antagonist, in healthy male subjects

**Authors:** Anthony Cahn, Simon Hodgson, Robert Wilson, Jonathan Robertson, Joanna Watson, Misba Beerahee, Steve C. Hughes, Graeme Young, Rebecca Graves, David Hall, Sjoerd van Marle, Roberto Solari

**PHARMACOKINETIC ASSAY**

***Microdose Intravenous Study.*** Aliquots (0.06 mL) of human plasma were analysed for total radioactivity by accelerator mass spectrometry (AMS). Samples were first prepared so that the carbon within the samples was harvested and converted to graphite. This involved a two stage process of sample combustion (oxidation) followed by graphitisation (reduction) as previously published [1]. The carbon analysis was performed using a Costech Elemental Combustion System (Model 4010) CHNS-O analyser (Valencia, CA, USA; supplied by Pelican Scientific Ltd. Chester, UK). The AMS used was a 250 kV single stage accelerator mass spectrometer (National Electrostatics Corp., Middleton, Wisconsin, USA). The instrument was operated via NEC proprietary “AccelNET” software on a Linux operating system. Post-acquisition data processing was performed using the NEC software “abc”. Further details about the operating conditions have been previously published [1].

Plasma concentrations of GSK2239633 were determined using a validated analytical method based on extraction by protein precipitation of 1 mL of human plasma with three volumes of acetonitrile. Both GSK2239633 and [^14^C]-GSK2239633, as a tracer, were extracted from 1 mL of human plasma using three volumes of acetonitrile containing non-labelled GSK2239633 at a concentration of 10 µg/mL, as an internal standard. The tubes were mixed by vortex and centrifuged for 10 minutes at approximately 3000 *g* prior to transfer of the supernatant to clean tubes. The dried extracts were reconstituted with 200 µL of water:acetonitrile (95:5, v:v) containing 0.1% formic acid, mixed and centrifuged at 3000 *g* for 30 minutes. The extract was then injected (100 µL) into a high performance liquid chromatography system utilising an Phenomenex Luna C18 (3 μ packing, 150 x 4.6 mm) column (Phenomenex Ltd., Macclesfield, UK) and eluted with a gradient of aqueous 0.1% formic acid and 0.1% formic acid in acetonitrile. GSK2239633 had a retention time of approximately 16.5 minutes and this was collected as discrete fractions into quartz tubes for further processing to harvest graphite and subsequent analysis by AMS as described above. The assay had a linear dynamic range of 1–500 pg/mL and quantification was performed against GSK2239633 spiked recovery standards based on demonstrated linearity with non-weighted regression.

***Single Oral Dose Study.*** Blood concentrations of GSK2239633 were determined using a validated analytical method based on extraction from a dried blood spot, with a 3 mm disc punched from a 0.015 mL sample on Ahlstrom 226™ card (PerkinElmer, Greenville, SC, USA). GSK2239633 was extracted using methanol (0.1 mL) containing an isotopically labelled [^2^H_3_, ^15^N_2_, ^13^C_1_]-GSK2239633 at a concentration of 10 ng/mL as an internal standard. The extraction tubes were shaken for 1 hour at room temperature before transferring the supernatant into clean tubes. The supernatant was injected (5 µL) into a high performance liquid chromatography system with an Acquity BEH C18 (1.7 μ packing 50 x 2.1 mm) column (Waters, Boston, Mass, USA) and eluted using a gradient of aqueous 0.1% formic acid and acetonitrile. GSK2239633 had a retention time of approximately 1 minute, with detection performed by tandem mass spectrometry on a Sciex 4000 (Applied Biosystems, Warrington, UK) using TurboIonSpray in positive polarity mode. Mass transitions from precursor ions were monitored for 368 m/z>374 m/z for GSK2396333 and 549 m/z>555 m/z for internal standard. The assay had a linear dynamic range of 10–10,000 ng/mL and quantification was performed using peak area ratios with 1/x^2^ weighted linear regression.

**REFERENCE**

1. Young GC, Corless S, Felgate CC, Colthup PV. **Comparison of a 250kV single-stage accelerator mass spectrometer with a 5MV tandem accelerator mass spectrometer - fitness for purpose in bioanalysis.** *Rapid Comms in Mass Spect* 2008, **22:**4035–4042.

# SUPPLEMENTAL TABLES

# Supplemental Table S1: Power model results of dose proportionality for GSK2239633 in the Single Oral Dose Study

| Parameter | Adjusted Mean Slope | SE (Logs) | 90% CI for Slope | %CVw |
| --- | --- | --- | --- | --- |
| AUC_0–10_ (ng.hour/mL) | 0.61 | 0.04 | (0.54, 0.68) | 19.80 |
| AUC_0–t_ (ng.hour/mL) | 0.65 | 0.05 | (0.56, 0.75) | 26.30 |
| C_max_ (ng/mL) | 0.76 | 0.08 | (0.63, 0.89) | 40.80 |
| CI: confidence interval; CVw: inter-subject variability; AUC_0–10_: area under the concentration-time curve from time 0 to 10 hours post-dose; AUC_0–t_: AUC from time 0 to last measurable concentration; C_max_: maximum observed concentration | | | | |

Supplemental Table S2: Summary of statistical analysis of food effect on blood GSK2239633 pharmacokinetic parameters in the Single Oral Dose Study

|  | GSK2239633 | | | | | |
| --- | --- | --- | --- | --- | --- | --- |
| Parameter | Comparison | Ratio | Fed geometric mean | Fasted geometric mean | 90% CI for ratio | %CVw |
| AUC_0–10_ (ng.hour/mL) | 1200 mg fed/1200 mg fasted | 3.08 | 4671.34 | 1518.86 | (2.63, 3.59) | 16.40 |
| AUC_0–t_ (ng.hour/mL) | 1200 mg fed/1200 mg fasted | 2.80 | 6523.58 | 2326.73 | (2.23, 3.53) | 24.70 |
| C_max_ (ng/mL) | 1200 mg fed/1200 mg fasted | 2.03 | 1408.56 | 695.41 | (1.46, 2.81) | 38.60 |
| CI: confidence interval; CVw: inter-subject variability; AUC_0–10_: area under the concentration-time curve from time 0 to 10 hours post-dose; AUC_0–t_: AUC from time 0 to last measurable concentration; C_max_: maximum observed concentration | | | | | | |

# SUPPLEMENTAL FIGURE LEGENDS

Supplemental Figure S1: Study design diagram of the Single Oral Dose Study.

Supplemental Figure S2: Box (median, 25 and 75^th^ percentile) and whisker (5 and 95^th^ percentile) plot for AUC_0–8_ (Panel A) and C_max_ (Panel B) for blood GSK2239633 in the Single Oral Dose Study.

# Supplemental Figure S1


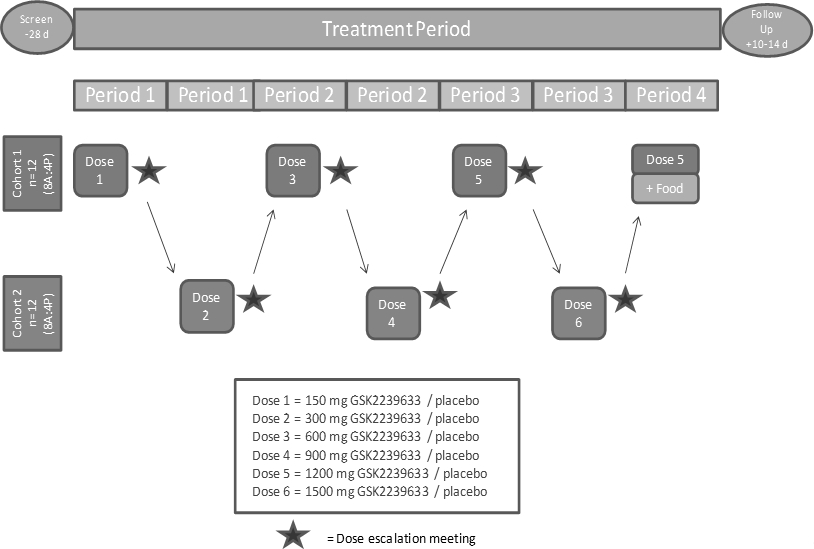


# Supplemental Figure S2A


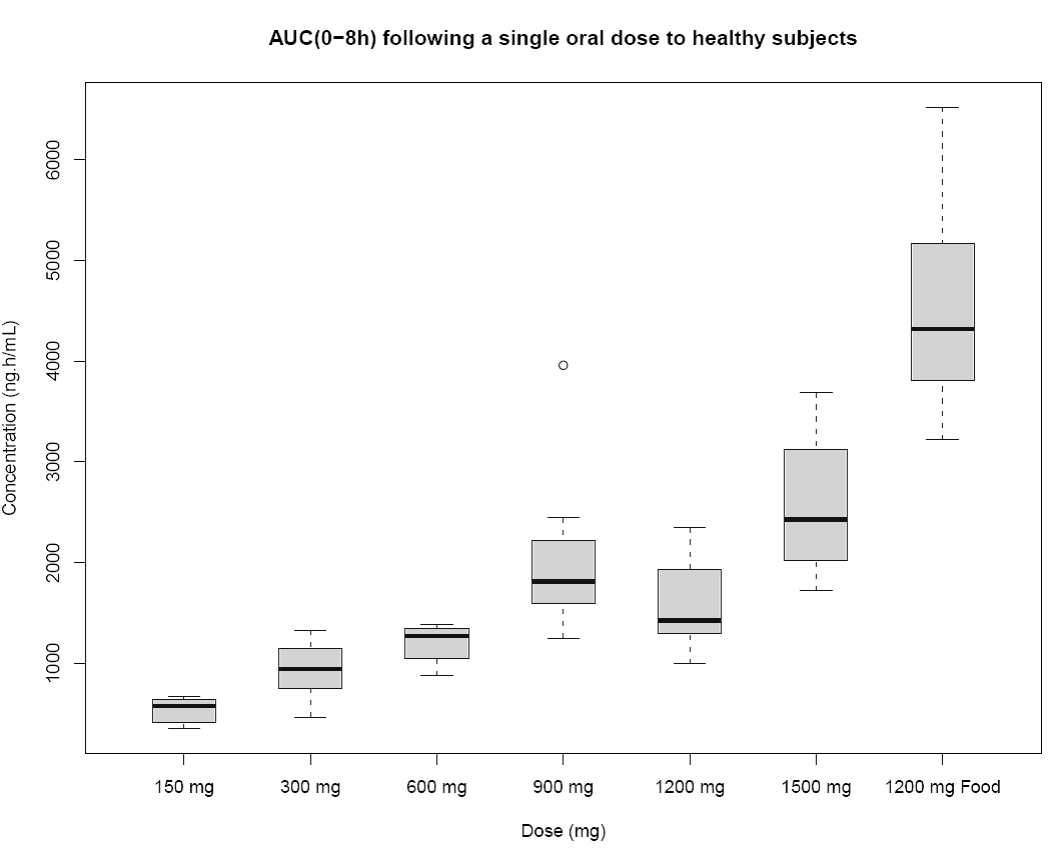


# Supplemental Figure S2B


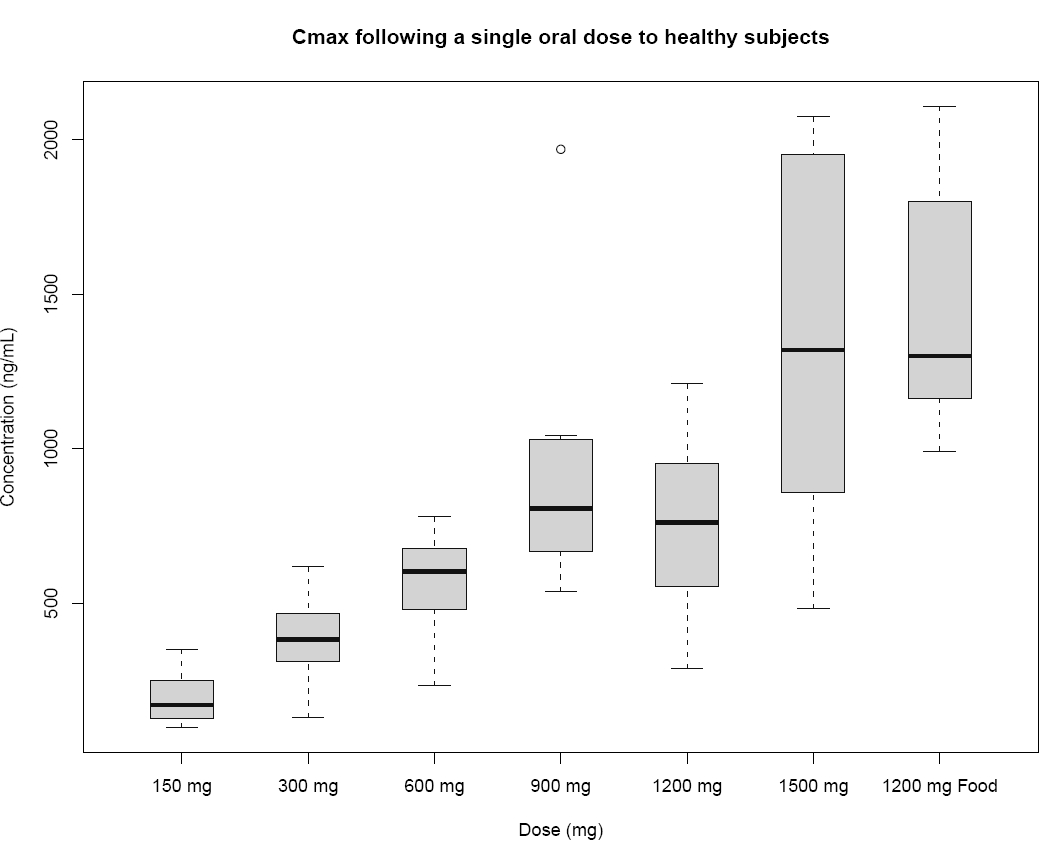

Supplement: Additional file 1 — Safety, tolerability, pharmacokinetics and pharmacodynamics of GSK2239633, a CC-chemokine receptor 4 antagonist, in healthy male subjects. Detailed information of the pharmacokinetic assays, 2 Tables, 2 Figures and figure legends. [file 2050-6511-14-14-S1.docx]
